# There is more to mental illness than negative affect: comprehensive temperament profiles in depression and generalized anxiety

**DOI:** 10.1186/s12888-018-1695-x

**Published:** 2018-05-10

**Authors:** Irina Trofimova, William Sulis

**Affiliations:** 1 Collective Intelligence Laboratory, Department of Psychiatry and Behavioral Neurosciences, McMaster University, Hamilton, Canada; 2 Hamilton, Canada; 3 Cayuga, Canada

**Keywords:** Major depression, Generalized anxiety disorder, Comorbid depression and anxiety, Age, Temperament, FET model

## Abstract

**Background:**

Temperament and mental illness are thought to represent varying degrees along the same continuum of neurotransmitter imbalances. A taxonomy of temperament could provide the basis for a new taxonomy of mental illness. Most popular models of temperament, being based heavily on emotionality traits, show very poor ability to discriminate between mental disorders. The main goal of this study was to examine whether a temperament model based on modern neurophysiology and possessing an extensive set of non-emotionality traits provides better discrimination between Major Depression (MD), Generalized Anxiety (GAD) and Comorbid MD and GAD, in comparison to emotionality-based temperament models.

**Methods:**

Using the Structure of Temperament Questionnaire, the temperament profiles of 687 individuals (396 clients of private psychiatric and psychological practice, and 291 control subjects) were compared across four adult age groups (18–24, 25–45, 46–65, 66–84).

**Results:**

MD and GAD appear to be accurately distinguished by the traits of Motor Endurance and Motor Tempo (much lower values in depression), and Neuroticism (much higher value in anxiety). Comorbids can be distinguished based on a significant decrease in the traits of Plasticity, Intellectual Endurance, Sensitivity to Probabilities, and increased Impulsivity. These effects seemed independent of age and gender.

**Conclusions:**

The results suggest the benefits of including non-emotionality-related traits and the usefulness of a functional approach to both taxonomy of temperament and classification of mental disorders.

**Electronic supplementary material:**

The online version of this article (10.1186/s12888-018-1695-x) contains supplementary material, which is available to authorized users.

## Background

A systemized classification of mental disorders is one of the key elements in providing assessment and treatment of these disorders [1]. The current schemes, *DSM-5* and *ICD-10,* use categories and criteria of mental illnesses that have been criticized for overlapping and ambiguous definitions and associated symptoms [2, 3]. One approach to addressing these criticisms comes from the study of temperament. Several authors have suggested that temperament and mental illness represent varying degrees along the same continuum of neurotransmitter imbalances in neurophysiological systems of behavioural regulation [4–13]. In fact, many studies have examined the relationships between temperament traits (such as impulsivity, sensation seeking, neuroticism, endurance, plasticity, sociability or extraversion) and various neurotransmitter and hormonal systems, (i.e. the very same systems implicated in mental disorders) [7, 14–21].

Research into the structure of temperament and the development of new DSM/ICD classifications mutually benefit since they both work on taxonomies related to the neurochemical regulation of behaviour [4–6, 22]. Much of the research in this area has, however, focused on temperament models and traits related primarily to emotionality, such as Negative Affect [6, 12, 23–25], Harm Avoidance [26–30], Neuroticism [4, 31–33] and Depressive Affective Temperament [34–36]. A recently proposed reconceptualization of the diagnostic categories for mood and anxiety disorders for the DSM-5 is also based on temperament models with dimensions of Negative and Positive Affects [3]. Affect-oriented temperament models appear to be very insensitive in differentiating between various types of mental disorders, especially between depression and generalized anxiety.

For example, anxiety disorders were associated with higher scores on Neuroticism/Negative Affect scales within Watson’s Positive/Negative Affects model [5, 6, 12, 22, 24], Mehrabian’s model [9], Cloninger’s model (TCI) [7], Trofimova’s Functional Ensemble of Temperament model (FET) [11, 37, 38], Akiskal’s model [7] and the Five Factor model [4, 10, 13, 33]. Neuroticism, however, appeared to be high not just in anxiety disorders but in many types of mental illness and therefore did not differentiate between mental disorders. For example, in addition to the association between high Neuroticism and anxiety disorders, Neuroticism/Negative Affect was also reported to have significant positive correlation with depression [4, 5, 8, 10, 12, 23, 24, 37–39]. Patients with histrionic personality disorder and major depression also scored higher on the Harm Avoidance scale (that is similar to Neuroticism) in studies using Cloninger’s TCI scales [14, 38–40].

These findings suggest that the scales measuring Neuroticism/Negative Affect in many current temperament models do not differentiate well between depression, generalized anxiety and other mental illnesses even in terms of the components of emotionality. In other words, according to these studies, Depression and GAD are more alike than they are different. Indeed in Watson’s quadripartite model [41], major depression and GAD lie within a single factor of “distress disorders”.

There is an interlocking between emotionality and functional (non-emotionality) aspects of behavioural regulation [19], but to base our psychological taxonomies only on emotionality aspects appears overly simplistic. The *DSM-5* considers a broad range of non-emotional symptoms: fatigue, poor attention and memory, dysfunction in sleep, appetite, psychomotor retardation, agitation, lethargy or restlessness, most of which have a connection to temperament. Nevertheless, non-emotionality aspects of behavior have received less attention in both studies of temperament and mental illness. A few studies have investigated the coupling between non-emotionality temperament traits and mental illness, primarily focusing on scales related to Extraversion, Sensation/risk seeking or Self-Directedness, but none have studied scales related to dynamical aspects of behavior, or physical functioning.

The scales of Extraversion and Self-Directedness have shown a weak ability to differentiate between different mental disorders, such as depression and GAD. Studies using the Five Factor model of personality reported a decrease in extraversion in depression and an increase of extraversion in mania [31–33]. Some studies have found associations of low Extraversion/Positive Affect with depression [5, 23] and GAD [10]. Extraversion, however, appears to conflate several traits having different psychophysiological etiologies: impulsivity, sociability, social-verbal tempo, physical endurance and sensation seeking (see [19] for a review) and therefore findings using the scale of Extraversion have limited applicability.

Studies using Gray’s Reinforcement Sensitivity model showed links of depression to lower Behavioral Activation and higher Behavioral Inhibition [9]. Studies using Cloninger’s Temperament Character Inventory (TCI) found reduced scores on the TCI Self-Directedness scale in depression [26, 30, 42, 43]. There are also reports of a correlation of GAD with behavioral inhibition [5, 10, 44] and a decrease in Self directedness [7, 14, 45].

Another temperament trait that is not related to emotionality was described as intellectual ergonicity [18], mental endurance [19, 20] or effortful control [46], known in psychology as sustained attention. Indeed, an inability to focus is one of the symptoms of GAD, and studies by Rothbart and Posner showed that there is an interaction between this temperament trait in healthy individuals and psychopathology.

One approach to a detailed taxonomy of emotionality and non-emotionality aspects of behaviour is provided by the neurochemical Functional Ensemble of Temperament (FET) model [18–20, 47]. The FET model was developed during a revision of the Structure of Temperament Questionnaire (STQ) model proposed by Rusalov 35 years ago (see [18] for a review). Using human subjects, he and his colleagues measured EEGs, evoked potentials, absolute thresholds in visual, auditory, and tactile modalities, strengths of excitation and mobility in auditory and visual modalities, performance under caffeine, problem solving in deterministic and probabilistic conditions, the speed of solving problems using a variety of intellectual tests, and other psychophysical tests. This research resulted in the first activity-specific model of temperament linking temperament traits to the functional components (aspects) of activities and separated physical-motor from social-verbal traits of temperament [18, 48, 49].

Trofimova revised Rusalov’s STQ model based upon an analysis of the functionality of neurotransmitter systems, Luria’s model of three neuroanatomic systems of behavioural regulation, and kinesiology models of human behaviour. This revision followed functional ecological, evolutionary and constructivist perspectives ([47, 50–53]) and suggested that the analysis of human taxonomy, as well as specific roles of neurotransmitters, should go hand in hand with an analysis of the structure and properties of human actions. This revision resulted in the integrated neurochemical Functional Ensemble of Temperament [18–20, 47] and also a Compact version of the STQ (STQ-77) reflecting the FET structure [11, 18, 54].

**Fig. 1:**
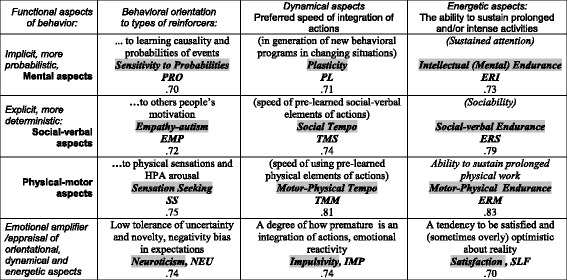
Description, abbreviations and alpha Cronbach of the scales of the Structure of Temperament Questionnaire (Compact, STQ-77) in the framework of the Functional Ensemble of Temperament (FET) model

The FET framework was proposed for use in the classification of mental disorders as well [11, 37–39], as it considers temperament in healthy people and mental illness as (correspondingly) weak and strong imbalances of the same neurotransmitter systems. This model organizes temperament traits and symptoms of mental illness in a 3 × 4 matrix categorized by functional aspects of human behaviour. The 12 components within the FET include: 9 systems (and traits) regulating formal functional aspects of behaviour (endurance, dynamic and orientational) each assessed in three domains (intellectual, physical and social), and 3 systems related to emotionality (Neuroticism, Impulsivity and Self-Confidence) (Fig. 1). The FET model suggests considering each symptom of mental illness from a functional perspective, as a process for constructing behavioural acts that has been compromised in some of its aspects.

Within the FET model, Plasticity is differentiated from Tempo traits, and refers to the ease in generating novel aspects of actions according to changes in situations, in integrating *new programs* of actions and in shifting between different tasks. In contrast to Plasticity, Tempo relates to the speed of *routine* elements of actions having lesser cortical involvement in comparison to Plasticity. Impulsivity reflects a tendency to act inadequately, in a reactive manner, without much planning or deliberation.

Throughout this paper, physical-motor and social-verbal will be referred to as simply physical and social respectively. The reader should keep in mind the original connotation. The definitions of traits are given in Fig. 1. Although not essential for the study reported here, it has been suggested that the nine non-emotionality traits are regulated by the monoamine (MA) (noradrenaline, dopamine, serotonin) and neuropeptide systems, whereas the three emotionality-related traits emerge as a dysregulation of opioid receptor systems that have direct control over MA systems (Additional file 1: Figure S1) [19, 20]. It is important to understand that the FET model suggests that there is no one-to-one correspondence between the neurotransmitter systems underlying temperament traits (or mental disorders). Instead, specific ensemble relationships between these systems emerge as temperament traits. The FET model suggests that the link between neurotransmitters and consistent characteristics of behaviour will manifest in patterns, similar to the manner in which spectroscopic patterns yield information about the structure of materials.

This study addresses the failure of previous temperament models to discriminate between major mental illnesses, especially in terms of emotionality and non-emotionality traits. If a temperament model has been carefully structured so as to be capable of responding to dynamical interrelationships between systems of behavioural regulation, then in the presence of mental illness, which presumably alters these relationships, these profiles should exhibit distinct patterns consistent with *DSM-5* symptoms of such illness. Therefore a systemic model or classification of mental illness should 1) have the ability to differentiate between components in a sufficiently detailed manner so as to be able to map empirically observed symptoms of mental disorders, and 2) have not only emotionality (affect)-related but also non-emotionality-related components reflecting the main functional aspects of behaviour.

The *objectives* of the study were:

1. Using the FET-based test, to examine the correspondence between the temperament profiles of patients with 3 active mental illnesses and the DSM/ICD symptoms of these disorders (Major Depression, Generalized Anxiety and Comorbid depression and anxiety).
2. To analyze the ability of the FET model to differentiate between these three disorders.
3. To analyze sex and age differences in the temperament profiles of people with these three disorders, as well as in healthy people.

### Hypotheses

Our *main (mental illness-related) hypothesis* was that, when an FET-based test of temperament (STQ-77) is used to assess temperament profiles, the following patterns in these profiles should differentiate between Control (healthy), Depression, Anxiety and Comorbid groups:

1. Major Depression: translating the DSM symptoms of fatigue and psychomotor retardation into a temperament frameworks, Depressed patients (compared to Controls) should (respectively) report lower scores on all three scales related to endurance (Physical, Social and Intellectual Endurance) and all three scales related to tempo.
2. Generalized Anxiety Disorder: Anxious patients (compared to Controls) should report lower scores on the scales related to orientation and sensitivity, (i.e. on Sensation Seeking and Sensitivity to Probability scales) but will not be significantly different from healthy people in their reported Physical Endurance and Tempo. Anxious patients will report lower sociability (i.e. Social Endurance), based on the reports of lower Extraversion in people with Anxiety [10].
3. Comorbidity: patients with Comorbid Depression and Anxiety will differ from Depression, Anxiety and Control groups by having significantly lower scores on the scales related to all aspects of activities, but especially scales related to behavioural integration (lower Physical Tempo, Social Tempo, Plasticity, higher Impulsivity). This was based on the fact that Comorbid depression and anxiety presents as a more serious disorder, with more severe symptomatology, a prolonged course, and diminished treatment response [55]. Thus it was hypothesized that Comorbid depression and anxiety should have the lowest scores in relation to all aspects of activities.
4. All patient groups will show significantly higher scores on the Neuroticism scale of the STQ-77, in comparison to the Control group, consistent with the existing literature [31–33, 37–39]. The scores of Anxiety patients are expected to be higher than those of the Depression group based upon the distinct emphasis applied to the symptom of worry in the criteria for GAD in the DSM/ICD classifications.

The effect of *age* on the expression of illness in temperament profiles was also examined in our study. It is well known that aging results in a progressive decline in physiological function, possibly influencing temperament and illness risk [56]. In normative samples the most consistent age-related differences in temperament traits are a decline in tempo-related characteristics [57] and sensation seeking [58]. Our *age-related hypothesis* was that age differences would be most significant in temperament traits related to dynamics of behaviour (tempo, plasticity and impulsivity) and sensation seeking in healthy people. It was not clear, however, if, or how, age affects the dynamical traits of temperament in anxious and depressed patients, and whether or not any age-related slow down amplifies any slow-down reported in patients with mental illness. It was also not clear if, or how, Sensation Seeking couples with mental illness and age. If age is a factor compromising the speed of behavioural integration and sensation seeking even more than mental illness, then we should observe statistically significant differences between the age groups of patients suffering from the same mental illness. The age groups were chosen for the study due to their correspondence to specific age-related functional tasks and stages of life: age 18–24 (transition from dependence on parents to independent living); age 25–45 (establishment of social, financial and psychological independence; building a professional career and setting up a family); age 46–65 (focus on career and exploration of personal interests; raising of children in their stage 1–2); age 66–85 (transition to retirement and subsequent retirement).

## Method

### Sample

The intake records of 687 Canadians aged 18–85, clients, patients in treatment and associates of a private psychiatric and psychological practice, Psychological Services 4018, were examined for this study. The practice serves Hamilton, Niagara Falls, Haldimand-Norfolk, Mississauga and Toronto areas, and the sample represents these five distant locations. The practice has its own Late Life Memory Clinic operating under the Haldimand War Memorial Hospital (Dunnville, Ontario) that provides testing and screening for dementia in clients and patients over 60. The final sample excluded subjects with dementia and protocols with poor validity scores (STQ Validity score > 13).

The demographic data of the sample (*N* = 687, M/F = 294/393, *Mean age/SD* = 43.75/19.27) is given in Table 1.

**Table 1:** Demographic characteristics of the sample

	Control	MD	GAD	Comorbid
All age groups	291	121	165	110
M/F, N	128/163	50/71	72/93	44/66
Mean_age_	43.33	57.35	33.58	45.18
SD_age_	22.07	11.48	18.38	14.45
Age < 25, N	74	0	66	0
M/F, N	30/44	0	29/37	0
Mean_age_	20.38	0	20.07	0/0
SD_age_	2.08	0	1.92	0/0
Age 25–45, N	88	36	62	58
M/F, N	35/53	17/19	30/32	29/29
Mean_age_	34.18	33.25	34.55	33.83
SD_age_	6.25	5.64	5.86	5.52
Age 46–65, N	70	42	37	52
M/F, N	30/40	16/26	13/24	15/37
Mean_age_	54.80	56.12	55.76	55.15
SD_age_	5.91	5.19	6.00	5.75
Age 66–84	59	43	0	0
M/F, N	33/26	17/26	0	0
Mean_age_	76.79	75.76	0	0
SD_age_	5.98	5.77	0	0
Range of tests scores:				
BDI	1–20	28–60	4–28	29–63
HDI	1–15	20–50	2–22	20–50
SCL-90D	1–20	31–52	13–20	32–52
BAI	1–21	6–21	22–41	22–42
STAI-S	20–39	26–48	50–78	60–79
SCL-90A	1–15	12–24	25–40	26–40

*BDI* Beck Depression Inventory-II [64], *HDI* Hamilton Depression Inventory [65], *SCL-90D(A)* Symptom CheckList-90, Depression (or Anxiety) scale [66], *BAI* Beck Anxiety Inventory [67], *STAI-S* State Trait Anxiety Inventory, S form [68]

### Procedure and measures

All participants of this study signed a consent form allowing the use of their intake forms for research purposes. Clients of our practice completed their intake forms (including questionnaires) during their initial contact with the Practice. Those patients whose protocols were selected for MDD, GAD or comorbid group were in an active state of illness, regardless of previous or subsequent history of illness. It is important to note that this study focused on the coupling between the acute state of mental illnesses and temperament profiles and did not extend to the topic of temperament differences in the subsequent course of illness. Thus we did not differentiate between the protocols of clients who did or did not experience subsequent remission of their illnesses. Diagnoses in the mental illness groups were made using *DSM-5* criteria based upon a structured DSM clinical interview, file review to obtain additional past history (if available), observation throughout the course of treatment (subjects were generally followed over the course of many weeks to years) plus the use of additional confirmatory tests. Cut-off scores for tests applied are listed in Table 1. These additional confirmatory tests varied between sites.. Subjects having a past history or current symptoms of Bipolar Disorder were excluded from this study.

Control subjects consisted of those clients who presented to the practice requesting counselling for problems such as marital and/or family dysfunction, problematic children or elderly parents, vocational choices, situational stress at work, etc. as well as healthy volunteers and their family members. Control subjects were all assessed to ensure that they did not meet the criteria for any DSM-5 diagnosis. Three control participants reported symptoms consistent with Adjustment Disorder (i.e., according to the DSM-5, a condition that lasted no longer than a month and should not be considered as a consistent behavioural pattern).

During either intake testing (for patients and clients) or research (for healthy participants), each person completed the Compact Structure of Temperament Questionnaire (STQ-77) [18, 50, 51]. The complete validation history can be found in [18, 50, 51, 54]. The STQ-77 consists of 77 statements, assigned to 12 temperament scales (6 items each) (corresponding to the FET model) and a validity scale (5 items, addressing social desirability bias), which are listed below. Results within the range of 15–20 on the validity scale were considered invalid as the respondents were likely to demonstrate a positive impression bias in their responses. Subjects responded according to a 4-point Likert scale format: (1) “strongly disagree,” (2) “disagree,” (3) “agree,” (4) “strongly agree.” The structure of this test, a description of the 12 temperament scales and their reliability indices as calculated for this data are given in Fig. 1.

### Statistical processing

- calculation of the descriptive scale statistics (Means, Standard Deviations, alpha Cronbach coefficient) for the STQ scales;
- factorial ANOVA for means on the STQ scales, with Diagnosis (Control, MD, GAD and Comorbid), Age and Sex (2 groups) as factors, to assess the effect of mental illness and compare it against the means of healthy participants. The effect of MD was assessed only in age-groups 25–85 (i.e. 3 older groups), the effect of GAD only in age-groups 18–65 (i.e. 3 younger groups) and the effect of Comorbid only in age groups 25–65 (two age groups in the middle of the age spectrum). These age limitations were due to the fact that there were not enough MD patients aged 18–25, not enough GAD patients over age 65 and not enough Comorbid patients in the youngest or oldest groups to form sub-samples for general comparison in our study.
- Since few sex differences among diagnoses were found, a factorial ANOVA was performed for the factors Diagnoses and Age using age groups as described in the paragraph above.
- Means for the two age groups in the middle age range (i.e. subjects aged 25–65) on the STQ scales were also submitted to factorial (Diagnoses, Age) ANOVA to examine the differences between temperament scores of the four diagnostic groups and their interactions with age.
- Sex differences were not a major object of this study, however they were examined for interactions with age, mental illness and temperament traits. To assess sex and age differences in temperament profiles the means of temperament scores of healthy participants were examined with factorial ANOVA (factors Age and Sex).

In all ANOVA processing, post-hoc comparisons were performed using both the Tukey and Fisher LCD tests with an alpha level of *.05*. The partial Eta-squared values (*η*^*2*^) were also calculated as an additional metric of effect size for all significant ANOVA contrasts.

The dataset supporting the conclusions of this article is available from McMaster University, Faculty of Health Sciences server: http://fhs.mcmaster.ca/cilab/DataDeprAnx.xls.

## Results

### Differences in temperament profiles associated with mental illness

#### Differences by scale in temperament profiles between patients with mental illness and control (healthy) groups

**Fig. 2:**
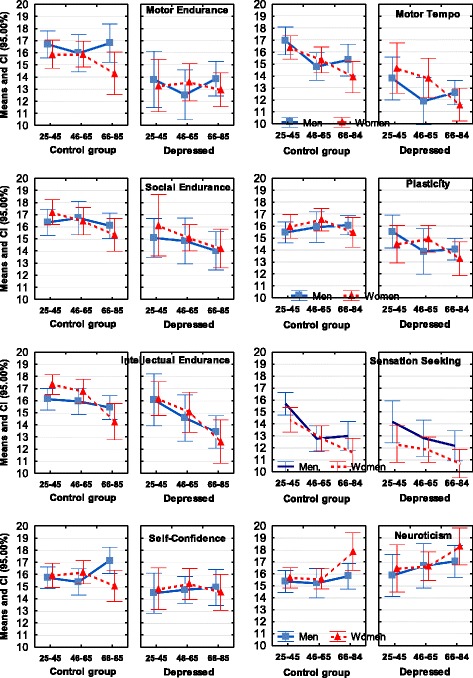
Means and Confidence Intervals (CI) of the temperament traits that had significant (at *p* < 0.001) effects of Major Depression

**Fig. 3:**
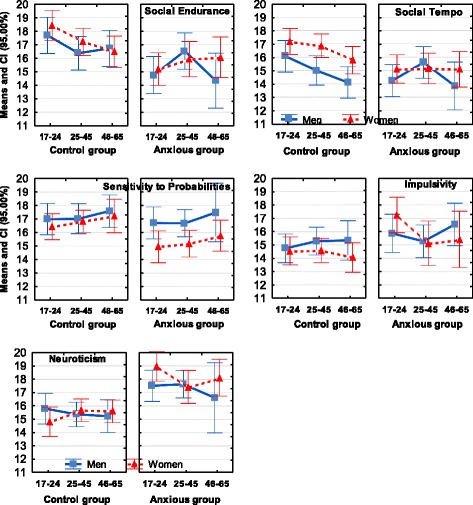
Means and Confidence Intervals (CI) of the temperament traits, which showed coupling with Generalized Anxiety

**Fig. 4:**
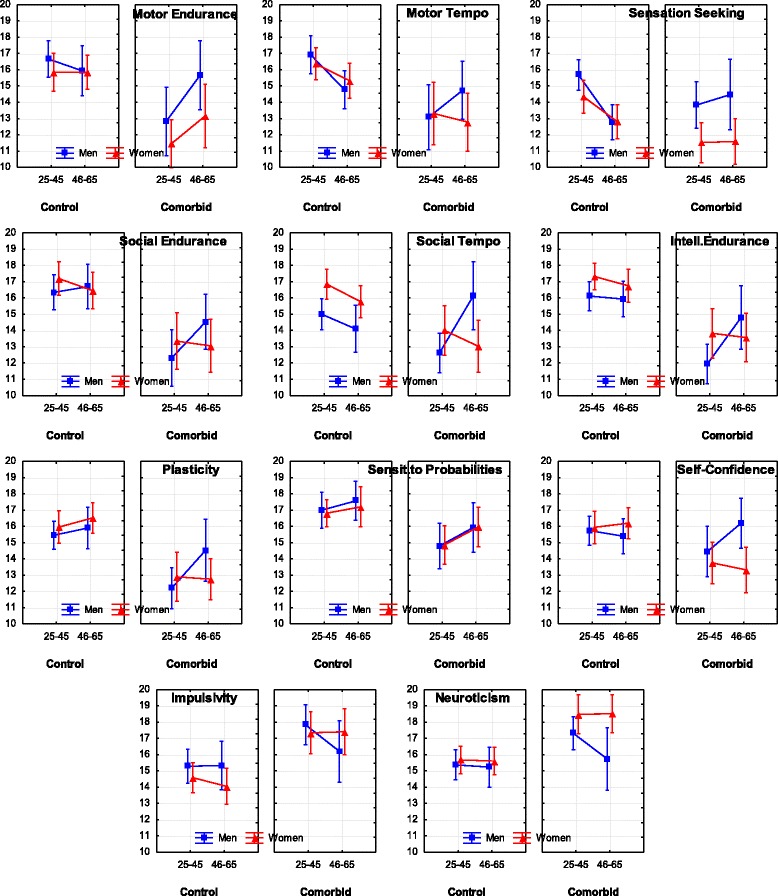
Means and Confidence Intervals (CI) of the temperament traits that had significant at *p* < 0.01 effects of coupling with Comorbid MD and GAD

Table 2 lists the means and effect sizes of factorial ANOVA for the factors Diagnosis and Age (i.e. without sex differences) whereas Figs. 2, 3 and 4 also show sex differences, in addition to the effect sizes of Diagnosis and Age.

**Table 2:** Means, SE (*M*_*SE*_) on the STQ-77 scales and effects of factorial ANOVA (factors Age and Diagnosis). Controls were compared to Major Depression (MD), Generalized Anxiety (GAD), and Comorbid (Cmrb) groups. Effect of MD was assessed in Age-groups 2–4 (*N*_Control_ = 217), effect of GAD – in Age-groups 1–3 (*N*_Control_ = 232) and effect of Cmrb - in Age groups 2–3 (*N*_Control_ = 158)

STQ-77	Controls: *N* = 217	MD *N* = 121	Effect of MD		GAD *N* = 165	Effect of GAD		Cmrb *N* = 110	Effect of Cmrb	
Scales	*M*_*SE*_	*M*_*SE*_	*F* (1,332)	*η* ^*2*^	*M*_*SE*_	*F* (1,391)	*η* ^*2*^	*M*_*SE*_	*F* (1,264)	*η* ^*2*^
Motor Endurance	15.94 _0.27_	13.29 _0.34_	33.53***	.091	15.71 _0.30_	0.35	.000	12.96 _0.49_	30.78***	.104
Motor Tempo	15.58 _0.24_	13.02 _0.34_	34.73***	.095	15.92 _0.29_	0.87	.002	13.26 _0.47_	24.97***	.086
Sensation Seeking	13.52 _0.23_	12.17 _0.27_	9.70**	.028	14.44 _030_	1.73	.004	12.55 _0.38_	8.33	.025
Social Endurance	16.45 _0.23_	14.85 _0.33_	13.62***	.039	15.53 _0.30_	17.82***	.044	13.13 _0.43_	51.92***	.164
Social Tempo	15.25 _0.22_	13.99 _0.33_	7.20**	.021	14.95 _0.26_	9.18**	.023	13.60 _0.39_	17.95***	.063
Empathy	16.55 _0.22_	16.45 _0.26_	0.054	.000	16.46 _0.22_	0.04	.000	15.48 _0.39_	4.40*	.016
Intellectual Endurance	15.93 _0.20_	14.32 _0.28_	14.17***	.041	15.72 _0.28_	6.44*	.016	13.70 _0.37_	57.75***	.179
Plasticity	16.11 _0.23_	14.47 _0.27_	20.84***	.059	15.34 _0.25_	2.68	.006	12.89 _0.35_	53.59***	.169
Sensitivity to Probabilities	16.87 _0.24_	16.17 _0.22_	2.04	.006	15.98 _0.26_	8.63**	.021	15.36 _0.32_	17.29***	.061
Selfconfidence	15.96 _0.21_	14.81 _0.28_	10.77**	.031	15.36 _3.72_	1.91	.005	14.12 _0.36_	16.29***	.058
Impulsivity	14.92 _0.23_	15.82 _032_	4.66*	.013	15.88 _0.29_	9.67**	.024	17.33 _0.35_	34.62***	.115
Neuroticism	15.83 _0.21_	16.93 _0.31_	7.13**	.021	17.87 _0.26_	53.07***	.119	17.80 _0.31_	35.91***	.119

Zeros at *η*^*2*^ values are omitted

* - *p* < 0.05, ** - *p* < 0.01, *** - *p* < 0.001

Temperament scores of the Depression group were significantly lower than scores of the Control group on all three endurance scales (Motor Endurance, Social Endurance, Intellectual Endurance), all three scales related to speed of integration of actions (Motor Tempo, Social Tempo, Plasticity), Sensation Seeking and Self-Confidence scales, and showed higher Neuroticism scores than the Control group. (Table 2, Fig. 2). The most extreme differences were observed on the scales of Motor-physical Endurance, Tempo and Plasticity.

Temperament scores of the Anxiety group were significantly lower than scores of the Control group on the scales of Social Endurance, Social Tempo and Sensitivity to Probabilities. The most significant difference was observed for the Neuroticism scale which was elevated in the Anxiety group (Table 2, Fig. 3).

The Comorbid group had significantly lower scores than the Control group on all three endurance-related (Motor, Social and Intellectual Endurance), dynamic scales (Motor and Social Tempo, Plasticity), Sensitivity to Probabilities and Self-Confidence, and higher scores than the Control group on the scales of Impulsivity and Neuroticism (Table 2, Fig. 4).

**Fig. 5:**
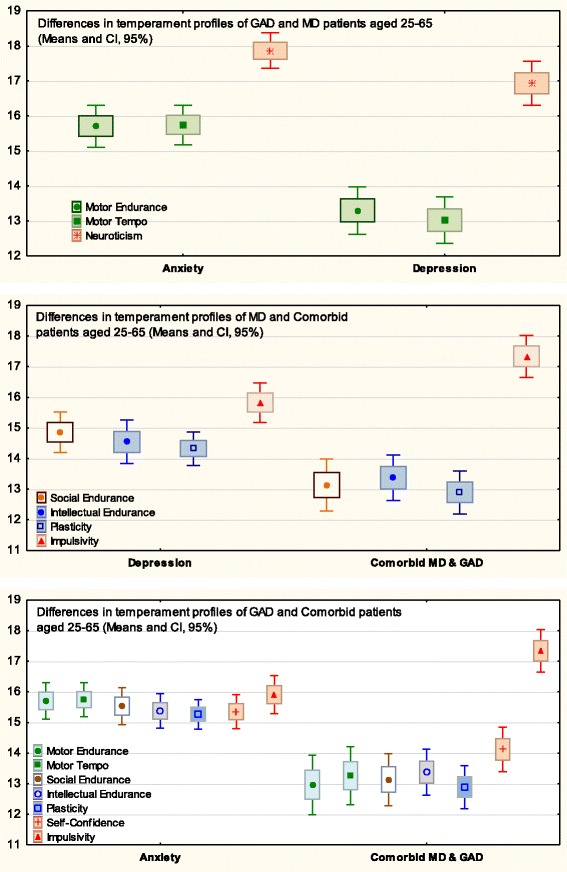
Means and Confidence Intervals (CI) of the temperament traits that differentiated between each pair of three diagnoses

Direct comparisons between the disorders using the Age2 and Age 3 groups yielded the following effects (Table 3, Fig. 5). Patients of the same age range diagnosed with Depression and Anxiety had significant differences on the scales of Motor Endurance and Tempo, with depressed patients scoring much lower than anxious patients. All three illness groups reported significantly higher Neuroticism, in comparison to Controls, however the scores of the Anxiety and Comorbid groups were significantly higher than the scores of the Depression group on this scale.

**Table 3:** Means, Standard Errors (*M*_*SE*_) on the STQ-77 scales and effects of Diagnosis (Major Depression (MD), Generalized Anxiety (GAD), Comorbid (Cmrb)) using factorial ANOVA (factors Diagnosis and Age) for temperament scores in groups aged 25–65

STQ-77	MD *N* = 78	GAD *N* = 99	Cmrb *N* = 110	MD vs GAD		MD vs Cmrb		GAD vs Cmrb	
Scales	*M*_*SE*_	*M*_*SE*_	*M*_*SE*_	*F* (1,173)	*η* ^*2*^	*F* (1,184)	*η* ^*2*^	*F* (1,205)	*η* ^*2*^
Motor-ph. Endurance	13.30 _0.49_	16.07_0.38_	12.96_0.49_	20.96***	.108	0.20	.001	23.19***	.101
Motor-ph. Tempo	13.26 _0.47_	16.15_0.38_	13.60_0.45_	16.23***	.086	0.31	.002	19.54***	.087
Sensation Seeking	12.67 _0.34_	13.55_0.34_	12.55_0.38_	2.17	.012	0.09	.000	2.90	.014
Social Endurance	15.27 _0.41_	15.90_0.37_	13.14_0.43_	0.84	.005	11.90**	.061	20.74***	.092
Social Tempo	14.74 _0.44_	15.10_0.32_	13.60_0.40_	0.07	.000	4.63*	.025	6.99**	.033
Empathy	16.67 _0.35_	16.10_0.35_	15.48_0.39_	1.08	.000	4.17*	.022	3.04	.014
Intellectual Endurance	15.43 _0.42_	15.89_0.35_	13.37_0.37_	0.04	.000	13.28***	.067	25.72***	.111
Plasticity	14.73_0.34_	15.51_0.31_	12.89_0.35_	2.17	.006	12.94***	.065	27.46***	.118
Sensitivity to Probabilities	16.44_0.44_	16.06_029_	15.36_0.32_	0.42	.002	3.95*	.021	2.63	.012
Self-confidence	14.87 _0.34_	15.74_0.37_	14.12_0.36_	2.53	.014	1.90	.010	8.66**	.040
Impulsivity	15.62 _0.38_	15.42_0.41_	17.34_0.35_	0.04	.000	10.39**	.053	11.01***	.051
Neuroticism	16.44_0.39_	17.55_0.33_	17.81_0.32_	4.75*	.027	7.58**	.040	0.26	.001

Zeros at *η*^*2*^ values are omitted

* - *p* < 0.05, ** - *p* < 0.01, *** - *p* < 0.001

Comparison of the profiles of MDD and Comorbid patients showed significantly lower scores on the cortically-regulated traits (i.e. scales of Intellectual Endurance, Plasticity, Sensitivity to Probabilities, Social Endurance) in Comorbid patients than MDD patients, and significantly higher Impulsivity scores in the Comorbid patients. Comorbid patients also had significantly lower scores, in comparison to the Anxiety group, on all three scales of Endurance, all three dynamic traits, and the Self-Confidence scale, and higher Impulsivity.

A summary of the results related to Diagnosis is presented in Fig. 7a and Table 2; the differences between diagnoses are shown in Fig. 5 and Table 3.

### Age-related differences in temperament profiles

**Fig. 6:**
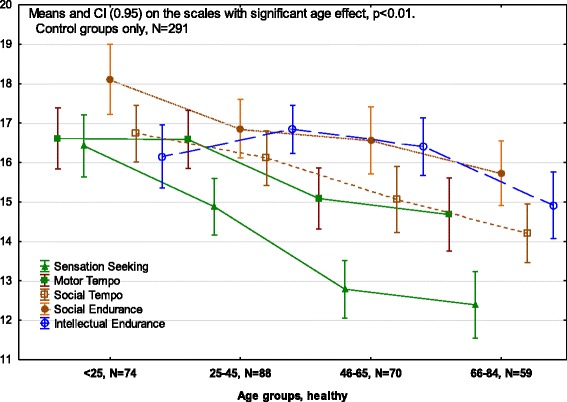
Age differences in temperament profiles of healthy participants

The most significant age effects (at *p* < 0.001) in the Control (healthy) group were found for the scales of Sensation seeking, Motor-Physical Tempo and Social-verbal Tempo, as well as a moderate effect (at *p* < 0.01) for the scale of Intellectual Endurance (Table 4, Fig. 6). The last effect was, however, not reflective of linear relationships and followed an inverted U shape, with the highest Intellectual Endurance (ability for sustained attention) reported not by the youngest group, but by the healthy participants in AgeGroups 2 and 3 (aged 25–45 and 46–65).

**Table 4:** Means, Standard Deviations (*M*_*SE*_) on the STQ-77 scales and effects of factorial ANOVA (factors Age and Sex) in the Control group, *N* = 291

STQ	Age < 25 *N* = 74	Age 25–45 *N* = 88	Age 46–65 *N* = 70	Age 66–85 *N* = 59	Effect of Age		Males *N* = 128	Females *N* = 163	Effect of Sex	
Scales	*M*_*SE*_	*M*_*SE*_	*M*_*SE*_	*M*_*SE*_	*F* (3,283)	*η* ^*2*^	*M*_*SE*_	*M*_*SE*_	*F* (1,283)	*η* ^*2*^
ERM	15.97 _.45_	16.17 _.41_	15.88 _.43_	15.66 _.59_	0.55	.006	16.77 _.34_	15.30 _.30_	11.85***	.040
TMM	16.61 _.39_	16.57 _.38_	15.09 _.39_	14.67 _.46_	7.11***	.070	16.13 _.31_	15.61 _.27_	3.08	.011
SS	16.42 _.39_	14.88 _.36_	12.79 _.37_	12.38 _.42_	24.60***	.207	14.64 _.31_	13.96 _.29_	6.02	.021
ERS	18.11 _.37_	16.85 _.37_	16.56 _.43_	15.73 _.41_	4.93**	.050	16.66 _.31_	17.04 _.29_	.119	.000
TMS	16.73 _.36_	16.11 _.35_	15.06 _.42_	14.20 _.37_	7.59***	.074	14.84 _.28_	16.24 _.26_	9.06**	.031
EMP	16.47 _.42_	16.73 _.32_	16.20 _.40_	15.98 _.41_	0.67	.007	16.20 _3.24_	16.80 _3.54_	2.38	.008
ERI	16.12 _.40_	16.84 _.31_	16.40 _.37_	14.92 _.42_	4.33**	.044	15.87 _.26_	16.40 _.27_	0.52	.002
PL	15.35 _.32_	15.76 _.34_	16.26 _.37_	15.80 _.34_	1.06	.011	15.64 _.24_	15.90 _.25_	0.39	.001
PRO	16.64 _.36_	16.87 _.33_	17.36 _.43_	15.80 _.47_	2.89*	.030	16.09 _.25_	15.82 _.25_	3.02	.010
SLF	15.85 _.31_	15.86 _.36_	15.86 _.35_	16.25 _.43_	0.15	.002	16.12 _2.75_	16.01 _3.33_	0.80	.003
IMP	14.62 _.37_	14.86 _.34_	14.60 _.44_	15.39 _.43_	0.65	.007	15.19 _.28_	14.57 _.27_	1.72	.006
NEU	15.22 _.40_	15.56 _.31_	15.44 _.35_	16.73 _.46_	3.19*	.046	15.55 _.26_	15.78 _.27_	1.37	.005

Zeros at *η*^*2*^ values are omitted

* - *p* < 0.05, ** - *p* < 0.01, *** - *p* < 0.001

Overall, Age showed no significant interaction effects with the factor Diagnosis suggesting that mental illness affects people of different ages in similar ways. The only strong (at *p* < 0.001) effect was found in differences between Comorbid and MDD patients aged 25–65 on the scale of Social-verbal Tempo: Comorbid patients reported low scores on these scales in both Age 2 and Age 3 groups, whereas MDD patients in the Age 2 group (25–45 years old) reported significantly higher Social Tempo, while the Age 3 (46–65) group in MDD patients reported Social Tempo which was even lower than in the Comorbid group (*F(1,184) =* 11.61; *η*^*2*^ = .059). This age-related drop in the scores of MDD patients likely contributed to weak (at *p* < 0.05) Age-Diagnosis interaction effects on this scale in MDD vs Control (*F(2,332) =* 3.81; *η*^*2*^ = .022, in 3 age groups) and MDD vs GAD (*F(1,173) =* 6.62; *η*^*2*^ = .037, in 2 age groups) comparisons.

There were a few other weak (at *p* < 0.05) Age-Diagnosis interaction effects: on the scale of Intellectual Endurance in comparing Comorbid and MDD patients (*F(1,184) = 4*.04; *η*^*2*^ = .021) and in compariing GAD and MDD patients (*F(1,183) = 6*.11; *η*^*2*^ = .034), patients in the Age 3 group estimated their ability to stay focused (i.e. Intellectual Endurance) significantly lower than other same-age participants, including patients with other diagnosis. Weak Age-Diagnosis interaction effects were found in comparisons between Comorbid and Control groups on the Sensation Seeking scale (SS) (*F(1,264) =* 4.29; *η*^*2*^ = .039) and on the Empathy scale, EMP (*F(1,264) = 6*.32; *η*^*2*^ = .023) - younger participants with mental illness had lower scores on these scales than their healthy peers. In comparisons between GAD and Control groups on the Social Endurance scale, anxious participants in the Age 2 Group reported higher scores than anxious participants in either Age 1 or 3 Groups, and this effect was especially profound in men (*F(2,391) =* 4.29; *η*^*2*^ = .021, 3 age groups, Fig. 3).

The results related to age differences in temperament traits are summarized in Fig. 6 and Table 4.

### Sex-related differences in temperament profiles

**Fig. 7:**
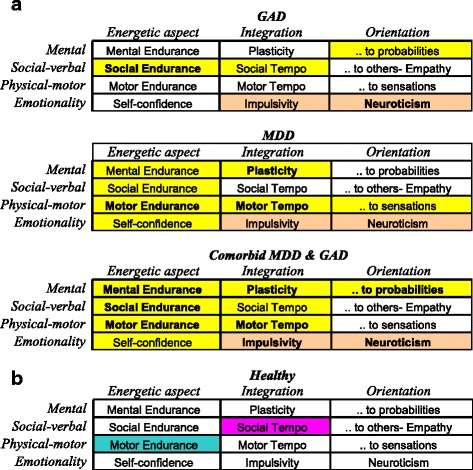
**a** Summary of the differential pattern of temperament profiles in three mental illnesses examined in this study. Bold fonts indicates the strongest ("signature") effects of a given diagnosis on specific A summary of the differential pattern of temperament profiles in three mental illnesses examined in this study. Bold fonts indicates the strongest ("signature") effects of a given diagnosis on specific aspects of behavioral regulation; yellow color – the scales showing significantly (*p* < 0.001) lower (and the pink color – higher) scores of mentally ill patients, in comparison to Controls. **b** Sex differences in temperament profiles of healthy participants: blue cell shows the trait that have higher scores in males, red cell – in females

In terms of sex differences, healthy men reported significantly higher scores in Physical Endurance compared to women, and lower Social Tempo (Table 4, Fig. 7b). There were no significant (at *p* < 0.01) Sex x Diagnosis interactions for Depression and Anxiety. There were weak Sex x Diagnosis interactions for the Comorbid group on scales of Sensation Seeking (*F(1,78) =* 4.277; *p =* .040; *η*^*2*^ = .021), Self Confidence (*F(1, 225) =* 5.690; *p =* .018; *η*^*2*^ = .028) (lower in women), and the scales of Social Tempo (*F(1,78) =* 6.572; *p =* .011; *η*^*2*^ = .031), Neuroticism (*F(1,78) =* 5.615; *p =* .019; *η*^*2*^ = .027) (higher in women).

## Discussion

### Differential temperament profiles associated with three mental illnesses

Our discussion mainly relates to the effects which are significant at *p* < 0.01 or lower.

As noted in the Introduction, previous attempts to find differential effects of Depression and Anxiety on temperament profiles relied mostly on emotionality traits (such as Positive-Negative Affect, Neuroticism). and this approach proved unsuccessful for differentiating between these major disorders. We argued that the use of a temperament model differentiating between physical, social and intellectual aspects of behavior, as well as between energetic, dynamical and orientational aspects, should result in a more nuanced discrimination of mental disorders. Indeed, when the FET framework was used to study temperament profiles, MDD, GAD and Comorbid depression and anxiety all showed distinct patterns of coupling with temperament profiles which can be distinguished one from another (Figs. 2, 3, 4, 5 and 7a, Tables 2 and 3). The structure of the FET model appeared very efficient in matching the “signature patterns” of temperament profiles of the clinical samples to the symptoms of the corresponding mental disorders as described in the DSM/ICD.

First, in line with our hypothesis and the DSM symptom of fatigue, MDD patients did indeed report lower scores on all three endurance-related scales compared to Controls and to subjects with Anxiety. Second, in line with the symptom of psychomotor retardation, MDD patients also had lower scores for all of the dynamic traits (Motor Tempo, Social Tempo and Plasticity).

This is consistent with the theory that diminished activity in serotonergic systems is linked to depression [56], and with the predictions of the FET model, since serotonin systems are major regulators of the energetic maintenance of behaviour [19, 20], and therefore there should be a decline in FET energetic traits. The serotonin system has long been implicated in the endurance aspects of activities although the relationship is complicated [59–61].

In contrast to these findings, Depression had less effect on the scales related to behavioural orientation to specific reinforcers (especially the scales of Empathy and Sensitivity to Probabilities). This finding points to possible difficulties in CBT if the focus is on cognitive constructs in depressed patients’ perception, i.e. orientational and not executive aspects of behaviour.

These differential effects show the benefits of distinguishing between endurance- and behavioural orientation-related traits when mapping symptoms of mental disorders.

In contrast to the FET model, the Cloninger model and the Clark-Watson model of Positive/Negative Affects lack endurance- and speed-related components (an exception is the scale of Persistence in the Cloninger model). Both Persistence in the Cloninger model and Plasticity in the FET model relate to facility in changing behavioral programs, each residing on the opposite poles of this characteristic. Differences in the items selected for these two scales were, perhaps, the reasons why studies using the Cloninger model did not show differences on the Persistence scale in MDD and GAD patients, in comparison to controls.

In line with the second part of our hypothesis and consistent with the DSM/ICD symptoms, GAD was associated with high Neuroticism scores. Moreover, Anxiety was also associated with significantly lower Social Endurance and Social Tempo (compared to Controls), and this is consistent with the commonly reported symptom of social withdrawal [10]. Compared to Controls, Anxiety patients also had significantly lower scores on the scale of Sensitivity to Probabilities (i.e. a low ability to comprehend the context and causal relationships between events). The differences in effects between MDD and GAD show the benefits of the activity-specific approach in temperament, distinguishing between traits related to physical, social and mental aspects of behavior. No other temperament model uses this differentiation but in the development of joint taxonomies for mental illnesses and temperament it might be crucial. The lower scores on Motor-Physical scales in patients with Depression might be considered as a signature in MDD profiles, while lower scores on the Social Endurance and Tempo might be indicators of Anxiety.

From a clinical standpoint, Comorbid depression and anxiety presents as a more serious disorder than MDD or GAD, with more severe symptomatology, a prolonged course and diminished treatment response [55]. Thus it was hypothesized that subjects with Comorbid depression and anxiety should report the lowest scores in relation to all aspects of activities. The results indeed showed significantly lower scores (compared to Controls) on 10 out of 12 scales (with the exception of Empathy and Sensation Seeking) in patients with a Comorbid diagnosis. The Comorbid MD and GAD group showed its own differential pattern in temperament profiles. Plasticity, sustained attention (measured as Intellectual Endurance), probabilistic processing (measured as Sensitivity to Probabilities) and impulse control (measured as Impulsivity) – are traditionally linked to the frontal cortex, and exactly these four traits showed very significant effects (Comorbid compared to Control). A differentiation between traits regulated by the frontal cortex (regulating implicit, probabilistic and complex aspects of behaviour), from traits regulating physical and verbal aspects of behavior was not proposed in either the Clark-Watson model or Cloninger model, but apparently such differentiation has practical value. The degree of negative change in these aspects of behavior differentiates between MDD, GAD and Comorbid depression and anxiety.

In our study, patients with a history of Bipolar Disorder, past or present, were specifically excluded and Comorbid subjects were treated separately. The very lage increase in Impulsivity in the Comorbid group is thought to represent a synergistic interaction between Depression and Anxiety. This is opposite to what would be expected in the presence of fatigue or psychomotor retardation in Comorbid patients, but consistent, however, with the FET hypothesis, which suggests that when executive capacities decrease, the plasticity of behaviour should be negatively affected, and impulsivity should increase. This in fact was observed. These results can be compared to the prediction of another temperament model, Gray’s Reinforcement Sensitivity Theory (RST). The RST describes two regulatory systems: the Behavioural Activation System (BAS) and the Behavioural Inhibition System (BIS) [16]. According to this model impulsivity occurs whenever there is an excess of BAS activation over BIS, while anxiety-neuroticism occurs whenever there is an excess of BIS over BAS. In Gray’s model, impulsivity cannot be a symptom of anxiety because anxiety and impulsivity arise in mutually exclusive states of BAS-BIS balance. Our results were not in line with the predictions of the RST, but were in line with the FET model. Moreover, several reports have also described elevated impulsivity in anxious and depressed patients [11, 37–39]. Thus an elevation of Impulsivity and a decrease in Plasticity may represent new symptoms of Comorbid depression and anxiety which warrant consideration for subsequent versions of the DSM/ICD. More general features differentiating between Major Depression and Comorbid depression and anxiety may be declines in the functions of cortical areas emerging as compromised plasticity, an inability to focus, impaired probabilistic thinking, and elevated impulsivity. This warrants further study.

Finally, consistent with the literature [31–33, 37–39], all three illness groups reported significantly higher scores on Neuroticism, but the Anxiety group reported the greatest scores on this scale. This converges with findings of higher Harm Avoidance in studies using the Five-Factor [12, 13, 31], Akiskal [34, 35], Mehrabian [9], and Cloninger models [14, 30, 42, 43] and Negative Affectivity using the Clark and Watson model [6, 10, 22, 24, 25]. This shows quite clearly that elevated Neuroticism by itself is a non-specific indicator of the three illnesses considered here although the degree of Neuroticism might be diagnostic. Very high Neuroticism might be a “signature” symptom of GAD or Comorbid depression and anxiety, and moderate Neuroticism might be merely a symptom of the presence of mental illness in general.

There are subtleties in the effects that these three disorders have on the Emotionality traits. As noted, Comorbid depression and anxiety was associated with much higher Impulsivity than the other two disorders while Anxiety was associated with much higher Neuroticism than the others two diagnoses. Depression and Comorbid depression and anxiety were both associated with lower scores in Self Confidence but the decline was greatest in the latter disorder.

The ability of the FET framework to differentiate between these three most common mental disorders demonstrates the value of the activity-specific approach in temperament research that could be employed for future classifications. Moreover, the current study has implications for modifying the classification OF and criteria for MD and GAD:

- DSM/ICD classifications can be implemented using the 3 × 4 structure of the FET framework, classifying all symptoms in terms of the endurance, tempo, or plasticity of actions, orientational aspects of behaviour and specific emotional dispositions. Symptoms could also be classified in terms of routine, habitual, physical and verbal aspects of behaviour (involving both basal ganglia and cortical networks) or behavioral elements related to more implicit contextual adjustments (with more involvement of the frontal cortex). Classifying symptoms within a formal matrix, instead of descriptive and overlapping lists, would result in a more compact and efficient taxonomy of mental disorders that would be in line with the structure of temperament in healthy individuals.
- Our study suggests that an additional symptom of social-verbal fatigue (appearing as social withdrawal and diminished speech volume), but not physical fatigue, should be added to the list of GAD symptoms.
- Our results suggest that the symptom of fatigue should be specified in at least three types (physical, social, intellectual) and not treated merely as a global non-specific factor.
- The diagnosis of Comorbid depression and anxiety can have its own specifyer-symptoms related to the profound negative changes in aspects of behaviour regulated by frontal cortical areas: significant loss of capacity for sustained attention (corresponding to the traits of Mental Endurance in the STQ and FET model [18–20], or Effortful Control in Rothbart and Posner [46] model); a significant loss of behavioural plasticity and of the ability to comprehend the context of a situation and anticipate future events (corresponding to the FET trait of Sensitivity to Probabilities), and high Impulsivity. High Impulsivity and low Plasticity can be considered as a pattern indicating the severity of mental disorders, such as Comorbid depression and anxiety. Differentiation between Major Depression and Comorbid depression and anxiety can be based on a simultaneous and more pronounced decline in the Comorbid state in cortical functions emerging as compromised plasticity, inability to focus and impaired probabilistic thinking, and elevated impulsivity.

### Age differences in temperament

The analysis of age differences in the temperament profiles associated with three mental disorders was conducted using four adult age groups. Surprisingly, there were practically no Age x Diagnosis interaction effects (i.e. there were no age differences in the way in which mental illness affected biologically-based aspects of behavioural regulation (temperament traits) of our participants). It appears that people of various ages suffer from mental illness in a similar fashion, and this was true for all three diagnoses. This is also in keeping with clinical findings that the expression of illness remains fairly consistent across the full age range.

There were, however, effects related to the factor of Age per se. Older age in normal subjects was associated with significantly lower scores for Physical Tempo, Social Tempo, Intellectual Endurance, Sensation Seeking, Social Tempo, and an increase in Neuroticism (Fig. 6). Lower Sensation Seeking in older age groups has been noted previously [58]. For Physical Tempo, Intellectual Endurance, lower scores were prominent only in the elderly age group, which parallels the fact that significant physical and intellectual decline does not occur until quite late in life [56, 57]. It is well known that there are differences in the presentation of anxiety and depressive illness in older age groups compared to young. The low occurrence of Depression in our youngest subsample (age 18–24) and the low occurrence of GAD in our oldest subsample (age 65–84) might be important observations by themselves.

As this was a cross sectional, rather than a longitudinal study, these results are only indirect evidence of the absence of noticeable differences across four age cohorts in the way in which mental illness affects temperament profiles.

### Sex differences in temperament

Gender appears to play a fairly minor role in the expression of these effects. Regardless of the presence of depression or anxiety, men reported significantly higher scores for Physical Endurance compared to women, consistent with well known physical (constitutional) differences between the sexes [58, 62, 63]. Women on the other hand reported significantly higher scores for Social Tempo than men (Fig. 7b). There were no significant Gender x Diagnosis interactions for Anxiety or Depression. This was quite interesting given the commonly held view that women are more emotionally vulnerable than men. In fact the only significant Gender x Diagnosis interactions occurred for Comorbid depression and anxiety involving Sensation Seeking, Self Confidence (lower in women) and Social Tempo, Neuroticism (higher in women). It is only in the presence of the more severe disorder that men and women begin to separate out significantly. This suggests that healthy men and women do not fundamentally differ in terms of their emotionality.

Sex differences in response to mental illness appeared to be related mostly to those traits on which men and women differ in their younger years, that is Physical Endurance (higher for men) and Social Tempo (higher in women). Indeed in Depression, younger men showed a greater decline in Physical Endurance compared to women, while women showed a greater decline in Social Tempo compared to men. These declines may be a direct result of the depression on behaviour regulation but one might speculate that they may also be due to the impact of depression on a person’s self judgment, which might make them overly critical of losses in the most prominent traits that participate in determining their sense of self. Men tend to see themselves as physically strong while women tend to view themselves are being more sociable and so these traits may be more vulnerable to negative judgment especially in younger people whose sense of self might not have stabilized.

Further studies will hopefully continue to assess the relationship between temperament profiles and symptoms of mental disorders, however the authors hope that current teams working on improving the DSM and ICD will consider the possibility of presenting both, mental disorders and healthy temperaments along a continuum, using the same components (formal descriptors or scales). We believe that the FET model, being supported by results in current psychophysiology [1, 19, 20, 47] provides a good start for developing a universal matrix for clinical and healthy psychological taxonomies of individual differences.

## Conclusion

The main focus of this study was to analyse the benefits of the FET framework for grounding a classification system for the diagnosis of mental illness. This framework is rooted in modern neurophysiology and at the same time reflects observations from clinical practice. This study investigated whether the FET framework, which possesses an extensive set of non-emotionality descriptors, might provide better discrimination between Major Depression, Generalized Anxiety Disorder and Comorbid depression and anxiety than existing temperament models. In order to assess this, direct head to head comparisons between disorders was carried out. The profiles of patients with these three mental disorders were compared across a broad range of adult age groups: age 18–65 for Anxiety, age 25–84 for Depression and age 25–65 for Comorbid anxiety and depression.

Our results showed that differentiating between emotionality and non-emotionality-related traits improved the sensitivity of temperament profiling and the specificity of symptoms of mental disorders. The results indicated that the three diagnoses could be differentiated in the following ways: 1) Major Depression was associated with significantly lower scores on Motor-physical Endurance. Motor-physical Tempo and Plasticity scales; 2) Generalized Anxiety Disorder was associated with significantly higher scores in Neuroticism and Social-verbal Endurance; 3) Comorbid depression and anxiety was associated with significant changes in scores on “cortical” scales: lower Mental endurance, Plasticity, Sensitivity to Probabilities and higher Impulsivity. These results are in line with the classic DSM/ICD symptoms of fatigue for Major Depression and worry in GAD, but they also suggested several useful insights for improving the DSM/ICD classifications of mental disorders, which we listed in our Discussion. As an important negative result, however, our study found that these three types of mental illness likely affect people in similar negative ways regardless of gender and age.

The detection of sex- and age-related differences in (mostly normative) profiles using the FET framework illustrates the benefits of separating out the dynamical and endurance-related aspects of behaviour, not only for clinical practice but for differential psychology generally. For example, analysis of age differences in temperament profiles in the Controls showed a lowering in Tempo and Sensation Seeking characteristics with age, but no changes in Endurance-related traits. Likewise, analysis of sex differences in the Controls showed that males have stronger Physical Endurance but slower Social Tempo than females.

Overall, the consistency between DSM symptoms and temperament traits within the FET model suggests that the FET matrix is a promising way to map functional aspects of behaviour for the formal classification of symptoms of mental illnesses. Seeking out matches between the structure of temperament and the structure of psychopathology might be a promising line of research, as suggested by several other authors [4–6] and points to the utility of using a functional approach to both the taxonomy of temperament and the classification of mental disorders.

The limitation of this study relates to the self-report nature of the STQ-77 used in this study. The self-report format is standard in temperament research and is also relied upon in deriving diagnoses using DSM/ICD criteria and in the SCID. The STQ Validity scale helped to minimize the effect of positive impression bias on data of this study. Moreover, the diagnosis was derived not just from a single interview and testing but from the observation during the treatment process as conducted by a licensed psychiatrist and clinical psychologist. The large sample size improves the power of the statistics, however, more research is needed to confirm the results reported here.

## Additional files

Additional file 1: **Figure S1.** The most consistent findings about the roles and interactions of neurotransmitter systems are integrated in the neurochemical model Functional Ensemble of Temperament (FET) (Trofimova, 2016, Trofimova & Robbins, 2016, Trofimova, 2018). (DOC 34 kb)

## Abbreviations

BAS: Behavioral Activation System
BIS: Behavioral Inhibition Systems
FET: Functional Ensemble of Temperament
GAD: Generalized anxiety disorder
MA: monoamines
MD: Major depression
RST: Reinforcement Sensitivity Theory
STQ: Structure of Temperament Questionnaire
TCI: Temperament and Character Inventory

## References

[1] Trofimova I, Robbins T, Sulis W, Uher J. Taxonomies of psychological individual differences: biological perspectives on millennia-long challenges. Philoso Trans R Soc, Biol. 2018; 10.1098/rstb.2017.0152. 10.1098/rstb.2017.0152 PMC5832678 29483338

[2] Follette WC, Houts AC (1996). Models of scientific progress and the role of theory in taxonomy development: a case study of the DSM. J Consult Clin Psychol.

[3] Insel TR (2014). The NIMH research domain criteria (RDoC) project: precision medicine for psychiatry. Am J Psychiatry.

[4] Ball SA, Tennen H, Poling JC, Kranzlen HR, Rounsaville BJ (1999). Personality, temperament, and character dimensions and the DSM-IV personality disorders in substance abusers. J Abnorm Psychol.

[5] Brown TA (2007). Temporal course and structural relationships among dimensions of temperament and DSM-IV anxiety and mood disorder constructs. J Abnorm Psychol.

[6] Clark LA, Watson D, Mineka S (1994). Temperament, personality, and the mood and anxiety disorders. J Abnorm Psychol.

[7] Heath AC, Cloninger C, Martin NG (1994). Testing a model for the genetic structure of personality: a comparison of the personality systems of Cloninger and Eysenck. J Pers Soc Psychol.

[8] Karam EG, Salamoun MM, Yeretzian JS, Neimneh ZN, Karam AN (2010). The role of anxious and hyperthymic temperaments in mental disorders: a national epidemiologic study. World Psychiatry.

[9] Mehrabian A (1995). Distinguishing depression and trait anxiety in terms of basic dimensions of temperament. Imagin Cogn Pers.

[10] Watson D, Naragon-Gainey K (2014). Personality, emotions, and the emotional disorders. Clin Psychol Sci.

[11] Trofimova I, Sulis W (2010). An investigation of temperament in adults with comorbid depression and anxiety. Adv Biosci Biotech.

[12] Weinstock LM, Whisman MA (2006). Neuroticism as a common feature of the depressive and anxiety disorders: a test of the revised integrative hierarchical model in a national sample. J Abnorm Psychol.

[13] Weiss A, Sutin AR, Duberstein PR, Friedman B, Bagby RM (2009). The personality domains and styles of the five-factor model are related to incident depression in Medicare recipients aged 65 to 100. Am J Geriatr Psychiatry.

[14] Cloninger CR (1986). A unified biosocial theory of personality and its role in the development of anxiety states. J Psychiatr Dev.

[15] Depue RA, Morrone-Strupinsky JV (2005). A neurobehavioural model of affiliate bonding: implications for conceptualizing a human trait of affiliation. J Behav Brain Sci.

[16] Gray JA (1982). The neuropsychology of anxiety: an enquiry into the functions of the septo-hippocampal system.

[17] Kagan J. Galen’s prophecy: temperament in human nature. Boulder: Westview Press; 1997.

[18] Rusalov VM, Trofimova IN (2007). Structure of temperament and its measurement.

[19] Trofimova I, Arnold MC (2016). The interlocking between functional aspects of activities and a neurochemical model of adult temperament. Temperaments: individual differences, social and environmental influences and impact on quality of life.

[20] Trofimova I, Robbins TW (2016). Temperament and arousal systems: a new synthesis of differential psychology and functional neurochemistry. Neurosci Biobehav Rev.

[21] Zentner M, Shiner R, editors. Handbook of temperament. New York: Guilford; 2012.

[22] Clark LA, Watson D (2006). Distress and fear disorders: an alternative empirically based taxonomy of the “mood” and “anxiety” disorders. Br J Psychiatry.

[23] Mineka S, Watson D, Clark LA (1998). Comorbidity of anxiety and unipolar mood disorders. Ann. Rev Psychol.

[24] Sellbom M, Ben-Porath YS, Bagby RM (2008). On the hierarchical structure of mood and anxiety disorders: confirmatory evidence and elaboration of a model of temperament markers. J Abnorm Psychol.

[25] Watson D, Clark LA, Carey G (1988). Positive and negative affectivity and their relation to anxiety and depressive disorders. J Abnorm Psychol.

[26] Nery FG, Hatch JP, Nicoletti MA, Monkul ES, Najt P (2009). Temperament and character traits in major depressive disorder: influence of mood state and recurrence of episodes. Depress Anxiety.

[27] Kampman O, Poutanen O (2011). Can onset and recovery in depression be predicted by temperament? A systematic review and meta-analysis. J Aff Dis.

[28] Kampman O, Poutanen O, Illi A, Setala-Soikkeli E, Met V (2012). Temperament profiles, major depression, and response to treatment with SSRIs in psychiatric outpatients. Eur Psychiatry.

[29] Lu X, Chen Z, Cui X, Uji M, Miyazaki W, et al. Effects of temperament and character profiles on state and trait depression and anxiety: a prospective study of a Japanese youth population. Depress Res Treat. 2012:604684 epub. 10.1155/2012/604684. 10.1155/2012/604684 PMC3432344 22957225

[30] Pelissolo A, Corruble E (2002). Personality factors in depressive disorders: contributions of the psychobiological model developed by Cloninger. L'Encéphale.

[31] Costa PT, Bagby RM, Herbst JH, McCrae RR (2005). Personality self-reports are concurrently reliable and valid during acute depressive episodes. J Affect Disord.

[32] Klein W, Kotov R, Bufferd SJ (2011). Personality and depression: explanatory models and review of the evidence. Ann Rev Clin Psychol.

[33] Kotov R, Gámez W, Schmidt F, Watson D (2010). Linking “big” personality traits to anxiety, depressive, and substance use disorders: a meta-analysis. Psychol Bull.

[34] Akiskal HS, Robins L, Barrett J (1989). Validating affective personality types. The validity of psychiatric diagnosis.

[35] Rihmer Z, Akiskal KK, Rihmer A, Akiskal HS (2010). Current research on affective temperaments. Curr Opin Psychiatry.

[36] Rovai L, Maremmani AG, Rugani F, Bacciardi S, Pacini M (2013). Do Akiskal and Mallya's affective temperaments belong to the domain of pathology or to that of normality?. Eur Rev Med Pharmacol Sci.

[37] Trofimova I, Christiansen J. Coupling of temperament traits with mental illness in four age groups. Psychol Rep. 2016;118(2). 10.1177/0033294116639430. 10.1177/0033294116639430 27154370

[38] Trofimova I, Sulis W (2016). Benefits of distinguishing between physical and social-verbal aspects of behaviour: an example of generalized anxiety. Front Psychol.

[39] Trofimova I, Sulis W (2016). A study of the coupling of FET temperament traits with major depression. Front Psychol.

[40] Kusunoki K, Sato T, Taga C, Yoshida Y, Komori K (2001). Low novelty-seeking differentiates obsessive-compulsive disorder from major depression. Acta Psychiatr Scand.

[41] Watson D (2009). Differentiating the mood and anxiety disorders: a quadripartite model. Ann Rev Clin Psychol.

[42] Hansenne M, Reggers J, Pinto E, Kjiri K, Ajamier A (1999). Temperament and character inventory (TCI) and depression. J Psychiatr Res.

[43] Mochcovitch MD, Nardi AE, Cardosa A (2012). Temperament and character dimension and their relationship to major depression and panic disorder. Rev Brazil Psiqiatrie.

[44] Fox NA, Pine DS (2012). Temperament and the emergence of anxiety disorders. J Am Acad Child Adolesc Psychiatry.

[45] Marco L (2013). Relationship between temperament and anxiety: a systematic review. Mediterr J Clin Psychol.

[46] Rothbart MK, Posner MI, Cicchetti D, Cohen DJ (2006). Temperament, attention, and developmental psychopathology. Handbook of developmental psychopathology.

[47] Trofimova I. Functionality vs dimensionality in psychological taxonomies, and a puzzle of emotional valence. Phil Tr Royal Soc, B. 2018. 10.1098/rstb.2017.0167. 10.1098/rstb.2017.0167 PMC5832691 29483351

[48] Rusalov VM. Biologicheskiye osnovi individual’no-psichologicheskih razlichiy (biological basis of individual psychological differences). Мoscow: NAUKA; 1979.

[49] Rusalov VM (1989). Motor and communicative aspects of human temperament: a new questionnaire of the structure of temperament. Pers Individ Diff.

[50] Trofimova I (2010). An investigation into differences between the structure of temperament and the structure of personality. Am J Psychol.

[51] Trofimova I (2010). Questioning the “general arousal” models. Open Behav Sci Psychol.

[52] Trofimova I (2016). Phenomena of functional differentiation (FD) and fractal functionality (FF). Internat J Des Nat Ecodyn.

[53] Trofimova I (2017). Functional constructivism: in search of formal descriptors. Nonlinear Dynam Psychol Life Sci.

[54] Trofimova I, Sulis W (2011). Is temperament activity-specific? Validation of the structure of temperament questionnaire – compact (STQ-77). Internat J Psychol Psychol Therapy.

[55] Coplan JD, Aaronson CJ, Pathangi V, Kim Y (2015). Treating comorbid anxiety and depression: psychosocial and pharmacological approaches. World J Psychiatry.

[56] Blazer D, Steffens D. Textbook of geriatric psychiatry. Washington, DC: APA Publishing; 2009.

[57] Birren J, Schaie W (2001). Handbook of the psychology of aging.

[58] Zuckerman M (1994). Behavioral expressions and biosocial bases of sensation seeking.

[59] Harrington ME (2012). Neurobiological studies of fatigue. Prog Neurobiol.

[60] Krishnan V, Nestler EJ (2010). Linking molecules to mood: new insight into the biology of depression. Am J Psychiatry.

[61] Struder HK, Weicker H (2001). Physiology and pathophysiology of the serotonergic system and its implications on mental and physical performance. Int J Sports Med.

[62] Costa PT, Terracciano A, McCrae RR (2001). Gender differences in personality traits across cultures: robust and surprising findings. J Pers Soc Psychol.

[63] Trofimova I (2015). Do psychological sex differences reflect evolutionary bi-sexual partitioning?. Am J Psychol.

[64] Beck AT, Steer RA, Ball R, Ranieri W (1996). Comparison of Beck depression inventories -IA and -II in psychiatric outpatients. J Pers Assess.

[65] Reynolds WM, Kobak KA (1995). Reliability and validity of the Hamilton depression inventory: a paper-and-pencil version of the Hamilton depression rating scale clinical interview. Psychol Assess.

[66] Derogatis LR, Savitz KL, Maruish ME (2000). The SCL-90-R and the brief symptom inventory (BSI) in primary care. Handbook of psychological assessment in primary care settings.

[67] Beck AT, Epstein N, Brown G, Steer RA (1988). An inventory for measuring clinical anxiety: psychometric properties. J Consulting Clin Psychol.

[68] Spielberger CD, Gorssuch RL, Lushene PR, Vagg PR, Jacobs GA. Manual for the state-trait anxiety inventory. Sunnydale: Consulting Psychologists Press; 1983.

