# Supplementary material for: There is more to mental illness than negative affect: comprehensive temperament profiles in depression and generalized anxiety
Source: BMC Psychiatry. 2018 May 10;18:125. doi: 10.1186/s12888-018-1695-x (PMC5946468; doi:10.1186/s12888-018-1695-x)
Supplement: Supplementary file 1 — Figure S1. The most consistent findings about the roles and interactions of neurotransmitter systems are integrated in the neurochemical model Functional Ensemble of Temperament (FET) (Trofimova, 2016, Trofimova & Robbins, 2016, Trofimova, 2018). (DOC 34 kb) [file 12888_2018_1695_MOESM1_ESM.doc]

**Supplemental material**

**Figure S1.** The most consistent findings about the roles and interactions of neurotransmitter systems are integrated in the neurochemical model Functional Ensemble of Temperament (FET) (Trofimova, 2016, Trofimova & Robbins, 2016, Trofimova, 2018).

| *Functional aspects:* | *Behavioral orientation to types of reinforcers … (NE+...)* | *Dynamical aspects*  Preferred speed of integration of actions *(DA+…)* | *Energetic aspects*  The ability to sustain prolonged and/or intense activities *(ACh, 5-HT+…)* |
| --- | --- | --- | --- |
| *Implicit, more probabilistic,*  **Mental aspects** | ***... to learning probabilities, PRO***  **NE+DA+ACh** | ***Plasticity vs. rigidity, PL***  **DA+5-HT** | ***Intellectual Endurance, ERI***  **NE, ACh** |
| *Explicit, more deterministic:*  **Social-verbal** | ***…to others: Empathy-autism, EMP***  **NE+OXY, VSP** | ***Social Tempo,***  ***TMS***  **DA+ PRL, OXY** | ***Social Endurance,***  ***ERS***  **5-HT+NP, OXY** |
| **Physical-motor** | ***…to sensations,***  ***SS***  **NE+NPY/SubP** | ***Motor Tempo,***  ***TMM***  **DA+PRL+NP** | ***Motor Endurance***  ***ERM,***  **5-HT+ACh, NP** |
| *Emotional amplifier/appraisal of orientational, dynamical and energetic aspects* | ***Neuroticism,***  ***NEU***  ***KOPr→NE-HPA***  ***KOPr > MOPr*** | ***Impulsivity,***  ***IMP***  ***DOPr→(DA, MOPr, BDNF, CREB)*** | ***Satisfaction,***  ***SLF***  ***MOPr→(5-HT,DA)***  ***MOPr >KOPr,SOM*** |

*Note*: 5-HT: serotonin; DA: dopamine; NE: noradrenalin; ACh: acetylcholine; GH: Growth Hormone; SOM: Somatostatin; PRL: prolactin; OXY: oxytocin; SubP: Substance P; NPY: Neuropeptide Y; KOPr, MOPr, DOPr: kappa-, mu- and delta-opioid receptors correspondingly.
